# Mammalian Ste20-Like Kinase and SAV1 Promote 3T3-L1 Adipocyte Differentiation by Activation of PPARγ

**DOI:** 10.1371/journal.pone.0030983

**Published:** 2012-01-26

**Authors:** Byoung Hee Park, Dae Soon Kim, Gun Woo Won, Hyun Jeong Jeon, Byung-Chul Oh, YoungJoo Lee, Eung-Gook Kim, Yong Hee Lee

**Affiliations:** 1 Department of Biochemistry, College of Medicine, Chungbuk National University, Chungbuk, Korea; 2 Department of Internal Medicine, College of Medicine, Chungbuk National University, Chungbuk, Korea; 3 Lee Gil Ya Cancer and Diabetes Institute, Gachon University of Medicine and Science, Incheon, Korea; 4 Department of Bioscience and Biotechnology, Sejong University, Seoul, Korea; Institute of Molecular and Cell Biology, Singapore

## Abstract

The mammalian ste20 kinase (MST) signaling pathway plays an important role in the regulation of apoptosis and cell cycle control. We sought to understand the role of MST2 kinase and Salvador homolog 1 (SAV1), a scaffolding protein that functions in the MST pathway, in adipocyte differentiation. MST2 and MST1 stimulated the binding of SAV1 to peroxisome proliferator-activated receptor γ (PPARγ), a transcription factor that plays a key role in adipogenesis. The interaction of endogenous SAV1 and PPARγ was detected in differentiating 3T3-L1 adipocytes. This binding required the kinase activity of MST2 and was mediated by the WW domains of SAV1 and the PPYY motif of PPARγ. Overexpression of MST2 and SAV1 increased PPARγ levels by stabilizing the protein, and the knockdown of SAV1 resulted in a decrease of endogenous PPARγ protein in 3T3-L1 adipocytes. During the differentiation of 3T3-L1 cells into adipocytes, MST2 and SAV1 expression began to increase at 2 days when PPARγ expression also begins to increase. MST2 and SAV1 significantly increased PPARγ transactivation, and SAV1 was shown to be required for the activation of PPARγ by rosiglitazone. Finally, differentiation of 3T3-L1 cells was augmented by MST2 and SAV1 expression and inhibited by knockdown of MST1/2 or SAV1. These results suggest that PPARγ activation by the MST signaling pathway may be a novel regulatory mechanism of adipogenesis.

## Introduction

The mammalian ste20 kinase (MST) pathway, also known as the Hippo pathway in Drosophila, is a potent regulator of organ size, and deregulation of this pathway leads to tumorigenesis [1]. The MST pathway negatively regulates proliferation and promotes cell death [1]. The MST pathway is composed of a serine/threonine (S/T) protein kinase MST1/2, a scaffolding protein Salvador homolog 1 (SAV1 or WW45), and a S/T protein kinase Large Tumor Suppressor (LATS), which are all homologs of the Drosophila proteins Hippo, Salvador and Warts, respectively.

There are two mammalian MST genes, MST1 and 2; the genes are almost identical in their kinase domains and exhibit a high degree of homology [2]. While MST1 is known to activate apoptosis in cell culture [3], [4], MST1 knockout mice showed only a mild phenotype in T cell physiology [5], [6]. The double knockout of MST1/2, however, results in embryonic lethality, suggesting a functional redundancy of MST1 and 2 [7]. Studies in Drosophila and mammalian systems have reported that SAV1 recruits LATS to MST to promote the phosphorylation of LATS by MST [8], [9] and that SAV1 is required for the correct cellular localization and function of MST [10]. Disruption of SAV1 in mice results in embryonic lethality with epithelial hyperplasia accompanied by defects in the terminal differentiation of various organs [10].

Recent studies have uncovered several downstream effectors of the MST signaling pathway [2]. Yes-associated protein 1 (YAP1), a transcriptional co-activator that is responsible for expression of multiple apoptosis-related genes, is phosphorylated and regulated by LATS, which in turn is phosphorylated and activated by MST [11], [12], [13]. MST1 activated by oxidative stress phosphorylates FOXO1/3a and inhibits the Akt-induced nuclear exit of FOXO1/3a [5], [14], [15]. Additionally, the phosphorylation of histone H2B by MST1 functions in chromatin compaction during apoptosis [16], [17]. The downstream effectors of the MST signaling pathway identified thus far are mainly regulators of cell proliferation and apoptosis and are involved in tumorigenesis. Those involved in cell differentiation have yet to be identified.

Peroxisome proliferator-activated receptor γ (PPARγ) is a member of the ligand-dependent nuclear hormone receptor family [18] and is a transcription factor that is activated by the insulin-sensitizing drugs, thiazolidinediones [19]. PPARγ is mainly expressed in adipose tissue [20] and stimulates adipogenesis of fibroblasts, such as 3T3-L1 preadipocytes [21], [22], through the activation of adipocyte gene expression [23], [24], [25].

PPARγ is phosphorylated and inhibited by extracellular-signal-regulated protein kinase 1 and 2 (ERK1/2), c-Jun N-terminal kinase and p38MAPK [26]. Additionally, PPARγ is reported to be regulated by direct binding of some protein kinases independent of phosphorylation; it is activated by direct binding of 3-phosphoinositide-dependent protein kinase-1 (PDK-1) [27] and inhibited by direct binding of MEK1 [28]. Despite these findings, the regulatory mechanisms controlling PPARγ activation during adipocyte differentiation are not fully understood.

In the course of identifying novel targets of the MST pathway, we identified a physical interaction between SAV1 and PPARγ that is stimulated by MST2. Here, we show that the association of MST2, SAV1 and PPARγ stimulates the transactivation of PPARγ and the differentiation of 3T3-L1 cells into adipocytes.

## Results

### MST2 and SAV1 interact with PPARγ

To identify novel SAV1-interacting proteins, we purified the SAV1 complex by immunoprecipitation from 293 cells overexpressing human SAV1 and/or human MST2. We chose MST2 because it has greater homology to Drosophila Hippo than MST1. From the mass spectrometric analysis of the SAV1 complexes, we obtained a list of proteins that included PPARγ2, a master regulator of adipogenesis [21] as well as PPARγ coactivator (PGC)-1β and Mediator complex subunit 1 (MED1), all of which are transcriptional coactivators for nuclear receptors, including PPARγ [29], [30]. SAV1, a scaffolding protein that functions in the MST signaling pathway, contains two type I WW domains that provide binding sites for other proteins that contain PPXY motifs [31]. We examined the amino acid sequence of PPARγ and found that it contains a PPYY motif spanning amino acids 113 to 116. Because of these findings, we decided to determine whether PPARγ is a novel target of the MST signaling pathway.

**Figure 1:**
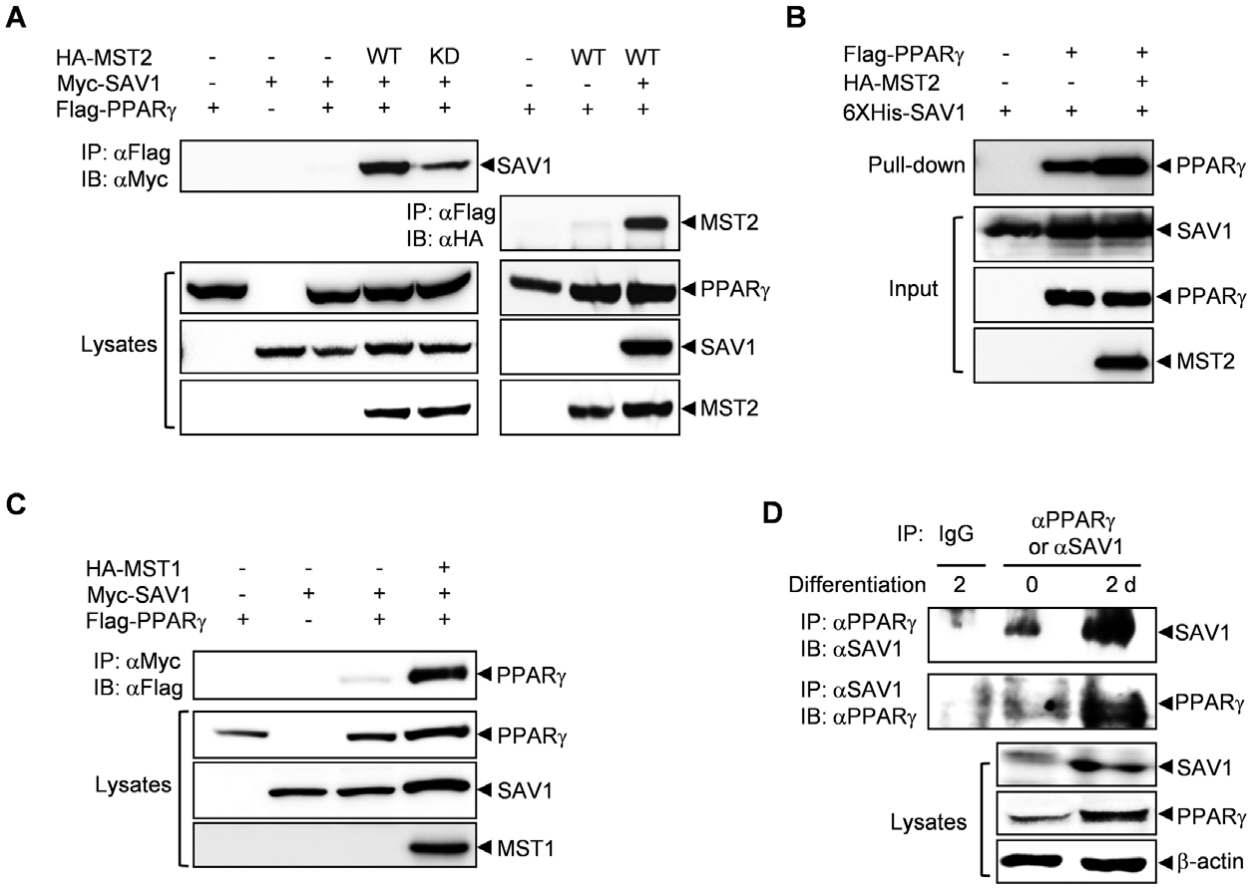
MST2 kinase stimulates the interaction of SAV1 and PPARγ. (A) The interaction between SAV1 and PPARγ was enhanced by MST2. 293 cells were transfected with HA-tagged wild-type (WT) or inactive (KD) MST2, Myc-SAV1 and/or Flag-PPARγ, and the interaction between PPARγ and SAV1 or MST2 was detected by immunoprecipitation with an anti-Flag antibody followed by immunoblotting with an anti-Myc or an anti-HA antibody. (B) Direct binding of PPARγ and SAV1 was confirmed by in vitro pull-down assay using recombinant hexahistidine-tagged SAV1 protein and PPARγ and MST2 proteins that were overexpressed in 293 cells. (C) MST1 also enhanced the interaction between SAV1 and PPARγ. The experiment was performed as described in (A). (D) The interaction between endogenous SAV1 and PPARγ was detected in 3T3-L1 adipocytes by immunoprecipitation with an anti-PPARγ antibody followed by immunoblotting with an anti-SAV1 antibody or inversely, immunoprecipitation with an anti-SAV1 antibody followed by immunoblotting with an anti-PPARγ antibody.

We first examined the association between SAV1 and PPARγ in 293 cells by performing co-immunoprecipitation experiments. As shown in Figure 1A, the interaction between SAV1 and PPARγ was very weak and almost undetectable when these two proteins were expressed together but was dramatically augmented by co-expression of MST2. Interestingly, the kinase-dead mutant of MST2 only slightly increased the interaction, suggesting that the catalytic activity of MST2 is required for full stimulation of the interaction between SAV1 and PPARγ. Interaction of MST2 and PPARγ was very weak unless SAV1 was co-expressed (Figure 1A), suggesting that SAV1 is playing a scaffolding role between MST2 and downstream targets like PPARγ. To determine whether interaction of PPARγ and SAV1 is direct, we carried out an in vitro pull-down assay. Recombinant hexahistidine-tagged SAV1 proteins was expressed in E. coli and purified with Ni-NTA bead. The recombinant SAV1 proteins immobilized on beads were incubated with 293 cell lysates over-expressing PPARγ and/or MST2, and the bound proteins were analyzed by immunoblotting. Direct binding of PPARγ and SAV1 was detected and it was increased by the presence of MST2 (Figure 1B). The interaction between SAV1 and PPARγ was also stimulated by co-expression of MST1 (Figure 1C). This result indicates that MST1 and MST2 have similar roles in adipogenesis.

We investigated the behavior of endogenous proteins. Co-immunoprecipitation experiments showed that the physical interaction of endogenous SAV1 and PPARγ was significantly increased 2 d after the initiation of differentiation in 3T3-L1 cells (Figure 1D). This increase in the interaction may result from a significant up-regulation of SAV1 and PPARγ protein expression in 3T3-L1 cells stimulated with differentiation media for 2 d.

### The WW domains of SAV1 and the PPYY motif of PPARγ are required for their interaction

**Figure 2:**
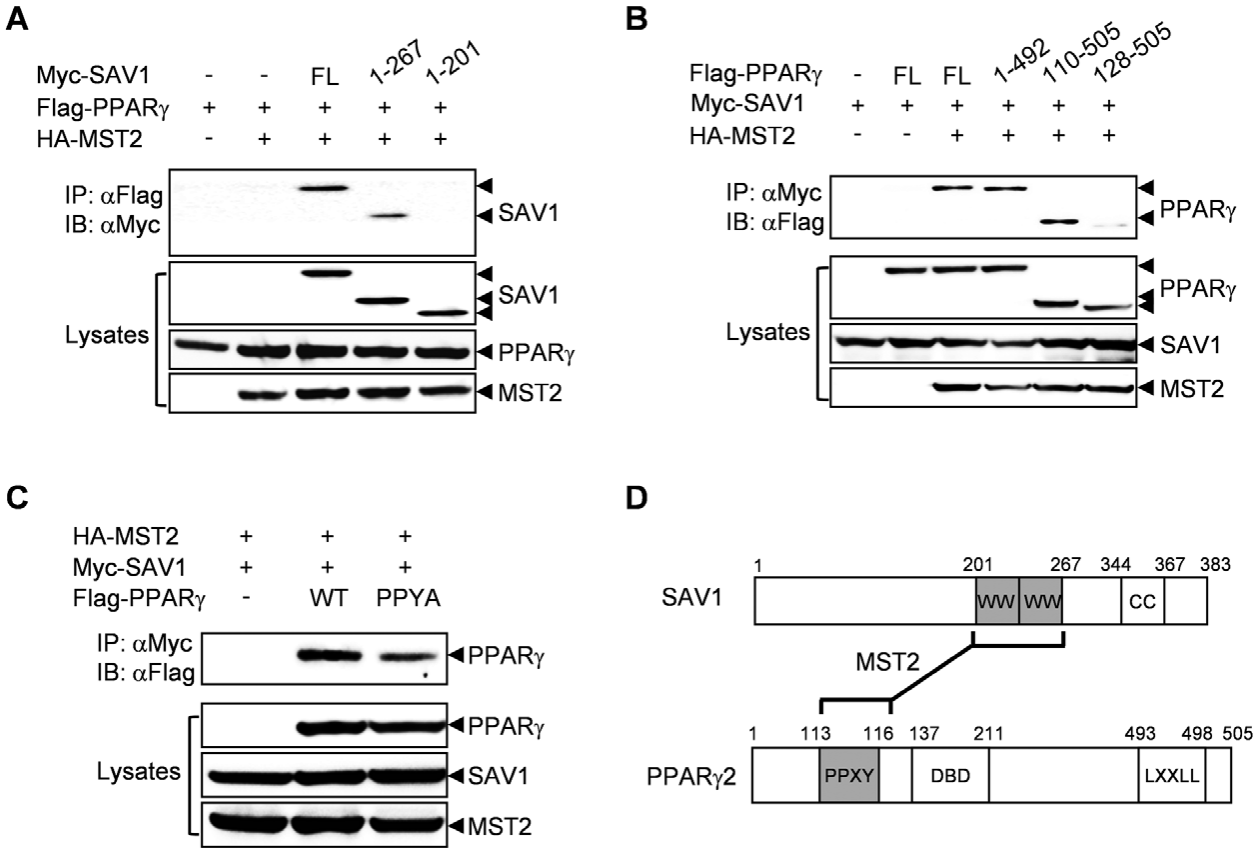
The WW domains of SAV1 and the PPYY motif of PPARγ are required for their interaction. (A) Myc-tagged SAV-1 deletion mutants were co-expressed with Flag-tagged PPARγ and HA-tagged MST2 in 293 cells. After 48 h, cell lysates were immunoprecipitated with an anti-Flag antibody and then immunoblotted with an anti-Myc antibody (upper panel). As a control, whole cell lysates were immunoblotted with the indicated antibodies (lower panels). (B) As above, the interactions of Flag-tagged PPARγ deletion mutants with Myc-tagged SAV-1 were analyzed. (C) Mutation of PPYY to PPYA in PPARγ decreases its interaction with SAV1. D. Schematic of the domain structure of SAV1 and PPARγ2.

To map the regions of SAV1 and PPARγ that are responsible for the interaction between these two proteins, we performed co-immunoprecipitation experiments using the deletion mutants of SAV1 or PPARγ. Full-length SAV1 and a SAV1 deletion mutant (1–267) containing the two WW domains bound to PPARγ, but a SAV1 deletion mutant (1–201) without the WW domains did not (Figure 2A). SAV1 was able to interact with PPARγ unless the PPYY motif of PPARγ was deleted (Figure 2B). Additionally, disruption of the PPYY motif in PPARγ2 by a point mutation (Y116A) significantly reduced the interaction with SAV1 (Figure 2C). These results clearly show that the WW domains of SAV1 and the PPYY motif of PPARγ are responsible for their physical interaction (Figure 2D).

### MST2 and SAV1 increase the levels of PPARγ by increasing its protein stability

**Figure 3:**
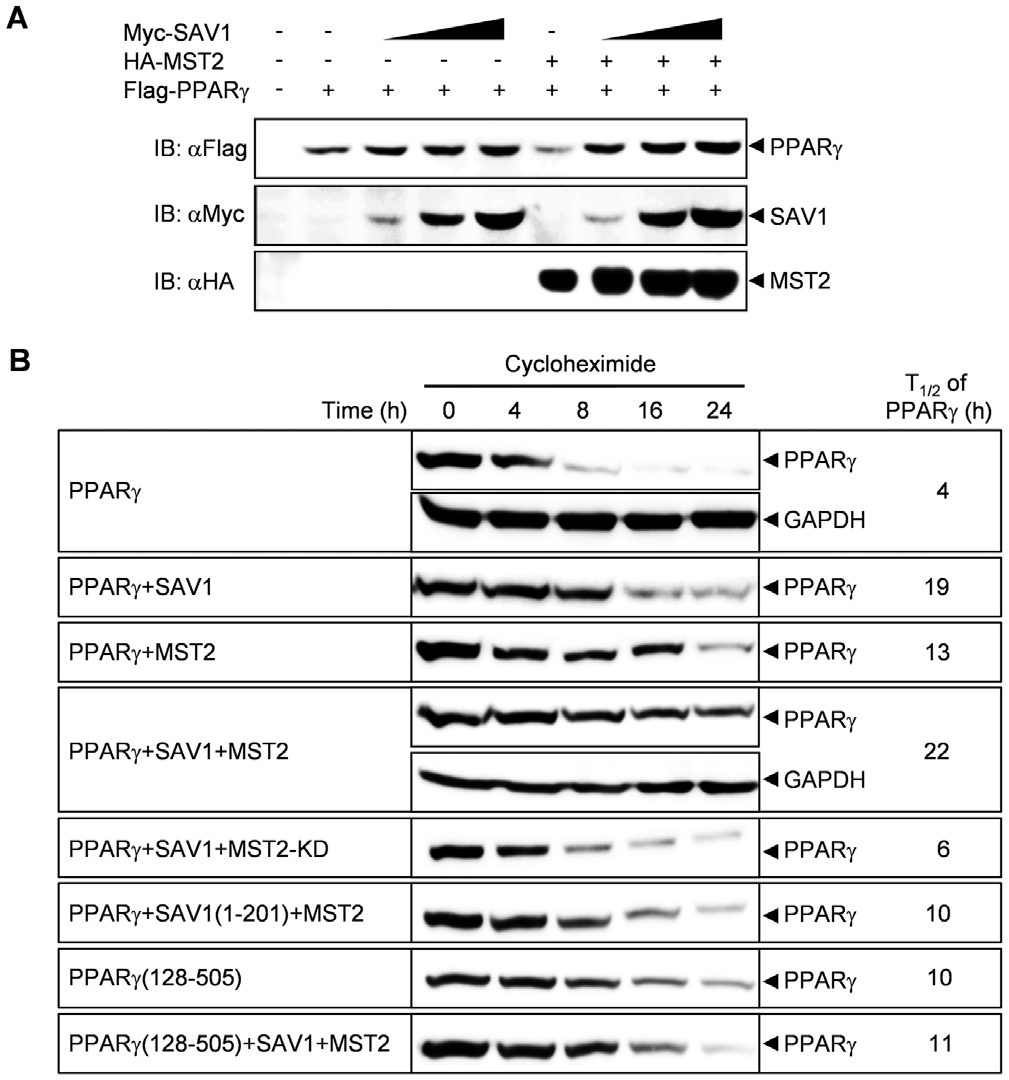
MST2 and SAV1 increase the levels of PPARγ by increasing its protein stability. (A) Increasing amounts (0.5, 1, 3 µg) of Myc-SAV1 were co-expressed with Flag-PPARγ and/or HA-MST2 in 293 cells. At 48 h after transfection, cell lysates were immunoblotted with the indicated antibodies. (B) Flag-tagged PPARγ or PPARγ(128–505) was co-expressed with HA-MST2, HA-MST2-KD (inactive mutant), Myc-SAV1, and/or Myc-SAV1(1–201) in 293 cells as indicated. Cell lysates were prepared at various times after treatment with 40 µg/mL cycloheximide and then immunoblotted with an anti-Flag antibody. The PPARγ protein expression was quantified and the half-life of PPARγ protein was calculated. Expression of GAPDH was detected as a loading control.

Because we noticed that PPARγ protein levels were always elevated upon the co-expression of SAV1 and MST2, we examined PPARγ protein expression more carefully. PPARγ protein levels were increased by SAV1 in a dose-dependent manner and were further augmented by MST2 (Figure 3A). To determine the half-life of PPARγ, 293 cells were transfected with combinations of PPARγ with SAV1 and/or MST2, and PPARγ protein levels were examined at various times after blocking protein translation with cycloheximide. PPARγ expressed alone degraded rapidly with a half-life of 4 h, but co-expression of SAV1 and/or MST2 significantly inhibited the degradation rate of PPARγ, extending the half-life up to 5.5-fold (Figure 3B). MST-KD, a kinase-inactive mutant did not enhance the PPARγ protein stability as efficient as wild-type MST (MST-WT). This is consistent with the result that MST2-KD is not as efficient as MST-WT in stimulating the physical interaction of SAV1 and PPARγ (Figure 1A).

A SAV1 deletion mutant (1–201) that is not capable of binding to PPARγ (Figure 2A) did not induce the stabilization of PPARγ as efficient as did wild-type SAV1. A PPARγ deletion mutant (128–505) that did not bind to SAV1 (Figure 2B) was not stabilized by co-expression of MST2 and SAV1. These data clearly indicate that the stabilization of PPARγ by MST2 and SAV1 requires a physical interaction between these proteins.

### The expression of PPARγ protein is dependent on MST2 and SAV1

**Figure 4:**
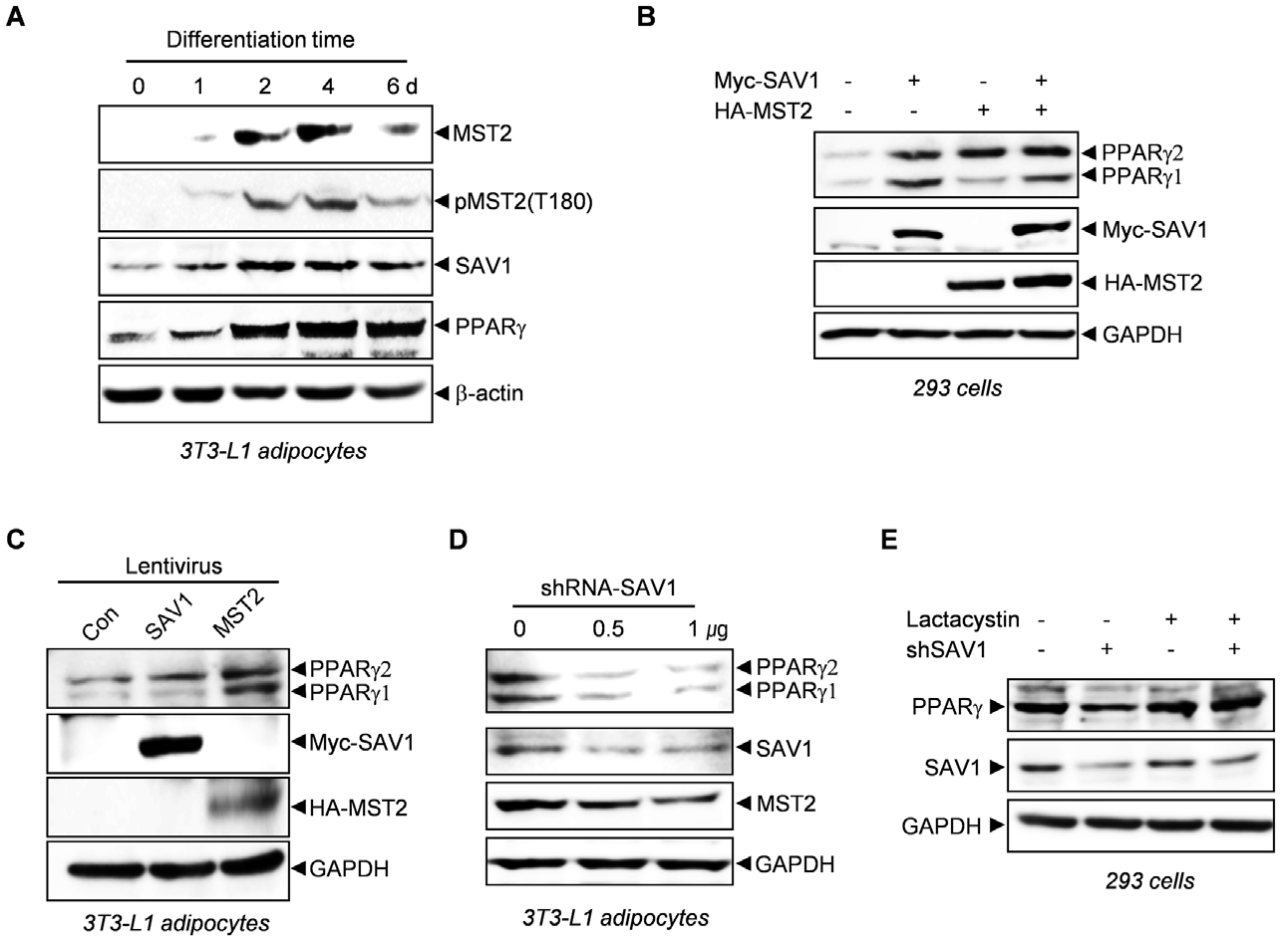
The expression of PPARγ protein is dependent on MST2 and SAV1. (A) The expression of SAV1 and MST2 increased during the differentiation of 3T3-L1 cells. Expression of the indicated proteins and phosphorylation of MST2 were analyzed with antibodies in the lysates of 3T3-L1 cells prepared at various times after induction of differentiation. (B) The levels of endogenous PPARγ proteins were increased by overexpression of MST2 and/or SAV1 in 293 cells. (C) The levels of endogenous PPARγ proteins were increased by lentivirus-mediated overexpression of MST2 or SAV1 in 3T3-L1 adipocytes. (D) The levels of endogenous PPARγ proteins were decreased by shRNA-mediated knockdown of SAV1 expression in 3T3-L1 adipocytes. (E) The degradation of PPARγ protein was proteasome-dependent. The decrease of PPARγ protein induced by knock-down of SAV1 expression was inhibited by 5 µM lactacystin, a proteasome inhibitor.

We then investigated the expression of endogenous MST2 and SAV1 proteins in differentiating 3T3-L1 adipocytes, in which PPARγ is of great importance. Pre-adipocytes showed low levels of MST2 and SAV1, but the expression of these two proteins increased at 2 d and stayed high as long as 6 d after the initiation of adipogenesis in 3T3-L1 cells (Figure 4A). The increase of MST1/2 kinase activity, as detected by a phospho-MST1(T183)/MST2(T180) antibody, similarly followed protein expression levels. A time-course of protein expression of MST2 and SAV1 matched well with that of PPARγ, supporting the idea that PPARγ expression may partially be dependent on MST2 and SAV1.

To confirm this, we increased or down-regulated the expression of MST2 and SAV1 and observed the expression of PPARγ. Overexpression of MST2 and SAV1 resulted in a significant increase of endogenous PPARγ protein in 293 cells and 3T3-L1 adipocytes (Figure 4B and C). Knockdown of SAV1 expression with shRNA inhibited the expression of endogenous PPARγ proteins in 3T3-L1 adipocytes (Figure 4D). Treatment of a proteasome inhibitor, lactacystin, reversed the down-regulation of PPARγ protein induced by knockdown of SAV1. This result shows that the degradation of PPARγ is proteasome-dependent (Figure 4E).

These results strongly indicate that an increase in PPARγ expression may be dependent on MST2 and SAV1 during the initial period of differentiation in 3T3-L1 adipocytes.

### MST2 kinase and SAV1 increase the transactivation activity of PPARγ

**Figure 5:**
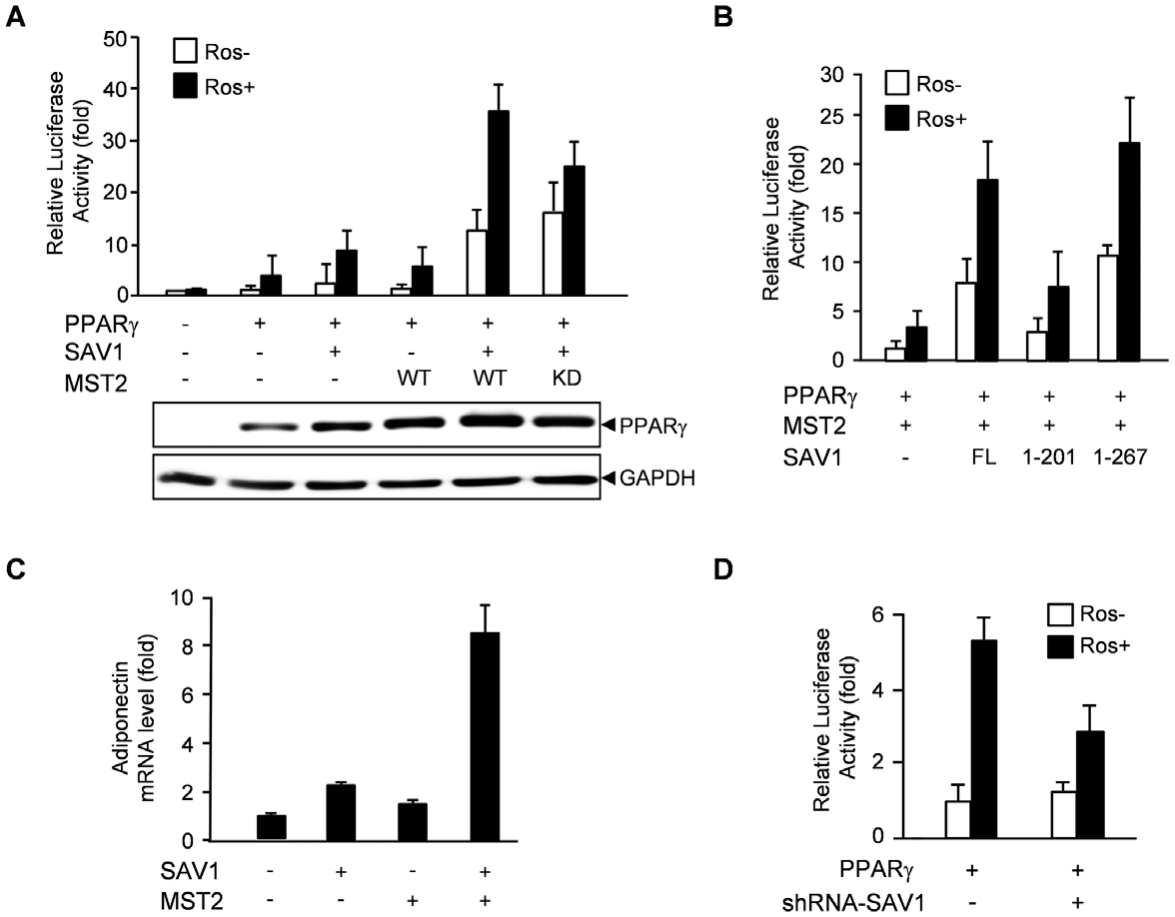
MST2 kinase and SAV1 increase the transactivation activity of PPARγ. (A) The transactivation activity of PPARγ increased upon co-expression of MST2 and SAV1. U2OS cells were transfected with a reporter gene, aP2-Luc, and pRL-TK along with PPARγ, SAV1 and/or MST2. Luciferase activity was assayed for the cells treated with (black bar) or without (white bar) 20 µM rosiglitazone. The lower panel shows expression of PPARγ and expression of GAPDH was detected as a loading control. (B) Full-length (FL) and deletion mutants of SAV1, SAV1(1–201) and SAV1(1–267) were transfected together with MST2 and PPARγ, and luciferase activity was assayed. (C) The mRNA level of adiponectin, a PPARγ target gene, was increased significantly by co-expression of MST2 and SAV1. Quantitative real-time PCR was performed with cDNA prepared from 3T3-L1 adipocytes transfected with MST2 and SAV1 as indicated. (D) The rosiglitazone-induced activation of PPARγ was inhibited by knockdown of SAV1 expression with siRNA against SAV1. All values are expressed as the mean ± SD (n = 3).

To determine the effect of MST2 and SAV1 on the transactivation activity of PPARγ, we performed a reporter assay using an aP2 promoter-dependent luciferase reporter in the presence of a selective PPARγ ligand, rosiglitazone (Figure 5A). Co-expression of SAV1, but not MST2, with PPARγ resulted in a 2-fold stimulation of the luciferase reporter activity compared with PPARγ alone in the presence or absence of rosiglitazone (Figure 5A). Consistent with the binding results, co-expression of both SAV1 and MST2 with PPARγ resulted in a dramatic 10-fold increase in the reporter activity compared with PPARγ alone in the presence or absence of rosiglitazone (Figure 5A). Expression of the kinase-dead mutant of MST2, along with SAV1 and PPARγ, showed a similar increase in the reporter activity in the absence of rosiglitazone as compared to wild-type MST2, but no further significant stimulation was seen in the presence of rosiglitazone (Figure 5A), suggesting that the kinase activity of MST may be required for the activation of PPARγ by its ligand. Immunoblotting of PPARγ shows that increase in PPARγ protein levels induced by SAV1 and/or MST2 was partly required but insufficient for full stimulation of PPARγ activity by co-expression of MST2 and SAV1 (Figure 5A, lower panel). This result suggests that stimulation of PPARγ transactivation activity by MST2 and SAV1 requires other mechanism, such as recruitment of co-activators or phosphorylation.

PPARγ was activated by a SAV deletion mutant, SAV1(1–267) that was shown to interact with PPARγ as efficiently as the full length SAV1 (Figure 5B). However, a SAV deletion mutant, SAV1(1–201) that is not capable of binding to PPARγ did not stimulate PPARγ activation (Figure 5B). These data clearly show that the activation of PPARγ by MST2 and SAV1 requires an interaction between these proteins.

We measured the effect of MST2 and SAV1 on mRNA level of adiponectin, one of the PPARγ target genes, by quantitative real-time PCR. Adiponectin mRNA expression was synergistically increased by MST2 and SAV1 (Figure 5C).

When we inhibit SAV1 expression with shRNA, the rosiglitazone-induced activation of PPARγ was significantly inhibited (Figure 5D). These data indicate that rosiglitazone may stimulate PPARγ activity by the MST signaling pathway.

### MST2 and SAV1 stimulate the differentiation of 3T3-L1 cells into adipocytes

**Figure 6:**
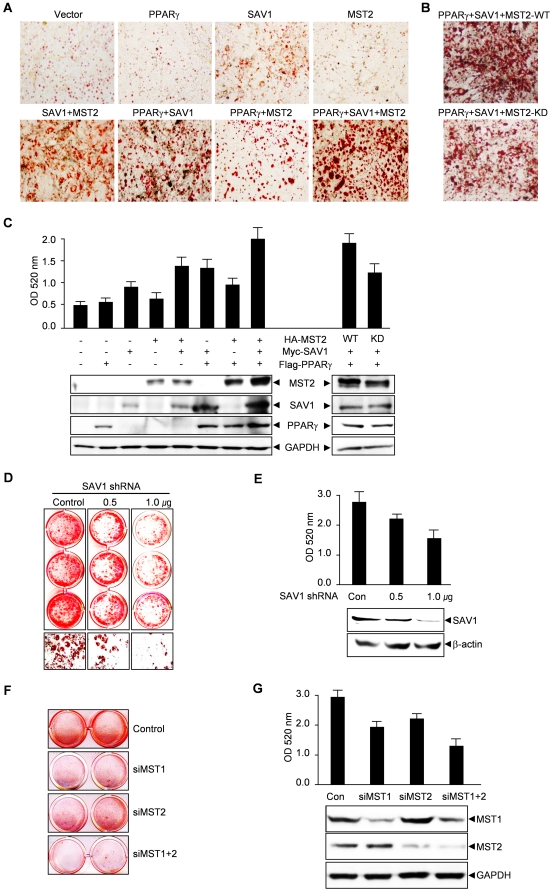
MST and SAV1 stimulate the differentiation of 3T3-L1 adipocytes. (A) Overexpression of MST2 and SAV1 stimulated differentiation of 3T3-L1 adipocytes. 3T3-L1 pre-adipocytes were transfected with PPARγ, SAV1 and/or MST2 expression plasmids and then stimulated with the minimal differentiation cocktail. After 10 d, cells were stained with Oil Red O and photographed using a microscope (200× magnification). (B) Kinase activity was required for the stimulation of differentiation of 3T3-L1 adipocytes by MST2. The experiment was performed as described in (A). (C) For quantification of samples from (A) and (B), Oil Red O stain was extracted and absorbances were measured at 520 nm. The lower panel confirms the expression of transfected proteins by immunoblotting. (D) Knockdown of SAV1 using shRNA inhibited differentiation of 3T3-L1 adipocytes. 3T3-L1 pre-adipocytes were transfected with a SAV1shRNA plasmid or a control plasmid and then stimulated with the full differentiation cocktail. After 10 d, cells were stained with Oil Red O and photographed. (E) For quantification of samples from (D), Oil Red O stain was extracted and absorbances were measured at 520 nm. The lower panel confirms the knockdown of SAV1 by immunoblotting. (F) Knockdown of MST1 or MST2 using siRNA inhibited the differentiation of 3T3-L1 adipocytes. The experiment was performed as described in (D). (G) For quantification of samples from (F), Oil Red O stain was extracted and absorbances were measured at 520 nm. The lower panel confirms the knockdown of MST1 or MST2 expression. All values are expressed as the mean ± SD (n = 3).

To assess a functional role for the MST signaling pathway in adipocyte differentiation, 3T3-L1 preadipocytes were transfected with various constructs and primed with insulin and a suboptimal concentration of rosiglitazone (Figure 6A). Cells transfected with SAV1, MST2 or PPARγ alone exhibited very low levels of adipocyte differentiation. However, expression of combinations of 2 proteins produced modest differentiation and expression of all 3 proteins showed a synergistic effect on adipocyte differentiation (Figure 6A and C). Differentiation was not stimulated by MST-KD as strongly as wild-type MST (Figure 6B and C), indicating a requirement for the kinase activity of MST2.

Additionally, to determine if MST2 or SAV1 is required for adipocyte differentiation, 3T3-L1 preadipocytes were transfected with SAV1 shRNA plasmid or MST1/2 siRNA and then stimulated with a full differentiation cocktail containing dexamethasone, IBMX and insulin. Down-regulation of SAV1 expression in 3T3-L1 cells resulted in a significant inhibition of adipocyte differentiation in a dose-dependent manner (Figure 6D and E). Knockdown of MST1 or MST2 inhibited adipocyte differentiation, and knockdown of both MST1 and MST2 showed a stronger inhibition of adipocyte differentiation (Figure 6F and G). These results show that the MST signaling pathway plays a major role in adipocyte differentiation of 3T3-L1 cells.

## Discussion

The MST signaling pathway is known to be an important player in the regulation of apoptosis and cell cycle control in Drosophila and mammalian systems and is believed to be a novel tumor suppressor pathway [1]. Many studies have focused on its role in tumorigenesis and cell proliferation. Our results show, for the first time, that PPARγ is an effector protein of the MST signaling pathway and provide evidence for a novel function of the MST signaling pathway in the terminal differentiation of adipocytes.

We showed that SAV1 functions as a scaffolding protein for the interaction of MST2 and PPARγ. Through mass spectrometric analysis of SAV1-interacting proteins, we found that SAV1 also binds to several transcriptional co-activators, such as PGC-1β and MED1, which are required for the activation of nuclear receptors, including PPARγ [29], [30]. Therefore, SAV1 may work as a scaffold to bring PPARγ close to important co-activators as well as MST2. Overexpression of MST2 and SAV1 induced a dramatic activation of PPARγ even in the absence of the ligand (Figure 5A and C), rosiglitazone, and this may result from the recruitment of coactivators to PPARγ by MST2 and SAV1. Inhibition of rosiglitazone-induced PPARγ activation by knockdown of SAV1 suggests that MST signaling may be activated by rosiglitazone and required for the process of PPARγ activation (Figure 5D).

The kinase activity of MST2 is partially required for stimulation of the SAV1-PPARγ interaction and stabilization and activation of PPARγ (Figure 1A, 3B, 5A, 6B). Several reports have shown that the catalytic activity of some protein kinases, such as PDK1 and MEK, is not required for the regulation of PPARγ activity by these kinases [28], [32]. MST2 as a protein kinase may work through two mechanisms: direct binding to PPARγ and phosphorylating PPARγ or its interacting co-activators or ubiquitin ligases. However, our preliminary attempts have not identified direct phosphorylation of PPARγ by MST2. Therefore, the catalytic activity of MST may be required to phosphorylate and regulate ubiquitin ligases or transcriptional co-activators. In addition, direct interaction of MST and SAV1 with PPARγ may be important in recruiting some regulatory proteins to PPARγ.

We showed that PPARγ protein levels were increased by co-expression of MST2 and SAV1, resulting from an increase in the stability of PPARγ (Figure 3, 4). This increase in protein stability may result from the down-regulation of ubiquitylation and proteasomal degradation of PPARγ (Figure 4E), and it partially explains the mechanism of PPARγ activation by MST2. It is of note that knockdown of SAV1 expression inhibited the expression and activation of PPARγ and the subsequent differentiation of 3T3-L1 adipocytes (Figure 4D, 5D, 6D). These results strongly support the idea that MST2 and SAV1 play important roles in adipogenesis.

It was reported that TAZ inhibits PPARγ and thereby blocks the differentiation of mesenchymal stem cells into adipocytes [33]. MST pathway was previously shown to inhibit epithelial-mesenchymal transition of some cancer cells stimulated by TAZ, which is phosphorylated and inhibited by LATS, a downstream kinase of MST [34]. Although we showed in this report that MST2 directly interacts with and activates PPARγ, it is possible that LATS and TAZ may have some role in the regulation of adipogenesis by MST.

It is possible that MST signaling performs different functions in the initial and the later phases of adipocyte differentiation. When confluent 3T3-L1 cells are exposed to differentiation stimuli, they undergo several rounds of cell division, called mitotic clonal expansion, followed by cell cycle exit to enter the terminal differentiation phase [35], [36]. The MST signaling pathway may be required to stop these initial cell divisions, as the protein levels of MST2 and SAV1 increased after 2 d of differentiation when cells are likely starting to exit the cell cycle. One study with SAV1 knockout mice suggested that MST and SAV1 are required for cell cycle exit and terminal differentiation of epithelial cells [10]. During this initial phase, MST signaling may not require PPARγ; rather, other cell cycle inhibitors, such as FOXO and YAP1, which have been previously shown to be effectors of the MST pathway, may be important [2]. In the later phase, MST2 and SAV1 may bind to and activate PPARγ to stimulate the expression of adipogenesis-related genes.

In conclusion, we have shown that MST2 interacts with and activates PPARγ through SAV1 and that MST2 and SAV1 together augment PPARγ-induced adipocyte differentiation. We propose that PPARγ activation by the MST signaling pathway may be a novel regulatory mechanism of adipogenesis.

## Materials and Methods

### Plasmids

Full length cDNAs for mouse PPARγ2, human SAV1 and human MST2 were obtained from the 21C Human Gene Bank (KRIBB, Korea) and subcloned into pCS4-3XMyc, -3XFlag or -3XHA mammalian expression vectors. The inactive MST2-K56R (MST2-KD) mutant was generated by site-directed mutagenesis and deletion mutants of PPARγ2, SAV1 and MST2 were made by PCR and confirmed by sequencing. A reporter plasmid, aP2-Luc, was kindly provided by Dr. J. H. Hong (Korea University, Korea). A SAV1 shRNA expression plasmid was obtained from Open Biosystems (RHS1764-9218744, USA). siRNAs for mouse MST1 and mouse MST2 were obtained from Bioneer (Daejeon, Korea). Human cDNAs of SAV1 or MST2 was subcloned into the lentiviral shuttle vector, and lentivirus was prepared and confirmed by Macrogen (Seoul, Korea).

### Antibodies

Antibodies against Myc (9E10; Santa Cruz Biotech), HA (12CA5; Roche Applied Science, Germany), Flag (M2; Sigma, USA), PPARγ (SC-7273; Santa Cruz Biotech, USA), MST2 (3952; Cell signaling, USA), phospho-MST1(Thr183)/MST2(Thr180) (#3681; Cell Signaling, USA), SAV1 (Abnova, USA), GAPDH (SC-166545; Santa Cruz Biotech, USA) and β-actin (LF-PA0209; AbFrontier, Korea) were used. Our antibody against SAV1 raised in rabbit with bacterially expressed human SAV1 protein (AbFrontier, Korea) was also used. Specific proteins were detected by incubating with horseradish peroxidase-conjugated anti-mouse (Pierce, USA) or anti-rabbit (Anaspec, USA) secondary antibodies.

### Cell culture and transfection

HEK 293 cells (CRL-1573; ATCC, USA) and HeLa cells (CCL-2; ATCC, USA) were maintained in Dulbecco's modified Eagle's medium (DMEM; Welgene, Korea) supplemented with 10% fetal bovine serum (FBS; Invitrogen, USA). U2OS cells (HTB-96; ATCC, USA) were maintained in McCoy's 5A medium (Welgene, Korea) containing 15% FBS. 3T3-L1 preadipocytes (CL-173; ATCC, USA) were maintained in DMEM containing 10% bovine calf serum (BCS; Invitrogen, USA). All media were supplemented with 100 units/mL penicillin-streptomycin (Sigma, USA) and all cells were maintained at 37°C in a humidified atmosphere with 5% CO_2_. Transfections were carried out using Lipofectamine Plus reagent (Invitrogen, USA) or Welfect reagent (Welgene, Korea). 3T3-L1 cells were transfected by Nucleofector II in solution V with program T-030 (Amaxa, Germany) and transfection efficiencies were always greater than 80%, as confirmed by GFP expression.

### Immunoprecipitation and immunoblotting

Cells were lysed with cold lysis buffer (50 mM Tris-HCl (pH 7.4), 120 mM NaCl, 1% NP-40, 12 mM β-glycerophosphate, 10 mM NaF, 0.5 mM PMSF, 5 µg/mL leupeptin, 5 µg/mL aprotinin, 1 µg/mL pepstatin, and 100 µM Na_3_VO_4_). Cell lysates were incubated with antibodies at 4°C for 2 h and complexes were subsequently retrieved with protein G-Sepharose beads (Amersham, UK). The immunoprecipitates were resolved by SDS-PAGE and transferred to polyvinylidene difluoride membranes (Millipore, USA). The membranes were immunoblotted with the indicated primary antibodies and the secondary antibody. The immunoblots were visualized by ECL kit (Elpis Biotech, Korea) and detected with LAS3000 system (Fuji, Japan).

### In vitro pull-down assay

Recombinant hexahistidine-tagged SAV1 protein was bacterially expressed and purified using Ni^2+^-nitrilotriacetic acid (Ni-NTA) beads (Invitrogen, USA). PPARγ and/or MST2 were over-expressed in 293 cells and the cell lysates were incubated with 6XHis SAV1 immobilized onto Ni-NTA beads at 4°C for 2 h in the lysis buffer that contained 5 mg/ml bovine serum albumin. The beads were rinsed with a washing buffer that contained 50 mM Hepes, pH 7.5, 150 mM NaCl, 1 mM EDTA, and 0.1% Tween 20. Proteins bound on the beads were separated by SDS-PAGE and analyzed by immunoblotting.

### Luciferase reporter assay

U2OS cells were transiently transfected with expression plasmids for PPARγ2, SAV1 or MST2, along with a luciferase reporter plasmid (aP2-luc) and a Renilla luciferase expression plasmid pRL-TK as an internal control. After 24 h, cells were treated with 20 µM of rosiglitazone (Caymann, USA) and cultured for an additional 24 h. The cells were lysed and measured for luciferase activity by the Steady Glo luciferase assay system (Promega, USA) using a luminometer (Berthold, Germany). Each transfection was performed in triplicate and the values were normalized to Renilla luciferase values.

### Quantitative real-time PCR

Total RNA was isolated from 3T3-L1 adipocytes 6 d after transfection with MST2 and/or SAV1 with TRIzol reagent (Invitrogen, USA) and further purified using RNeasy kit (Qiagen, Germany). cDNA was generated from 2 µg total RNA using Moloney murine leukemia virus reverse transcriptase (NEB, USA) and oligo(dT) primers. Quantitative real-time PCR to detect mouse adiponectin was carried out using QuantiTect SYBR Green PCR kit (Qiagen, Germany) in a Rotor-Gene RG-3000 cycler (Corbett Research, Australia). PCR conditions were: denaturating at 95°C for 10 min followed by 40 cycles of amplification with 10 s at 95°C, 15 s at 62°C, and 20 s at 72°C. GAPDH was used for normalizing the expression data. The primers used were as follows: mouse adiponectin forward primer, 5′-catcccaggacatcctggccacaatg-3′; reverse primer, 5′-ggcccttcagctcctgtcattccaac-3′; mouse GAPDH forward primer, 5′-gtgaaggtcggtgtgaacg-3′; reverse primer, 5′-ggttcacacccatcacaaac 3′.

### Adipocyte differentiation assay using Oil Red O staining

3T3-L1 cells were maintained in DMEM with 10% BCS. Two days after reaching confluence, cells were transfected by Nucleofector and were stimulated 24 h later with the differentiation cocktail for 2 d. Cells expressing the vector control, PPARγ, SAV1 and/or MST2 were treated with a minimal differentiation cocktail containing 10% FBS, 5 µg/mL insulin and 0.5 µg/mL rosiglitazone. Under these conditions, differentiation was virtually absent in vector-transfected cells. The 3T3-L1 preadipocytes were transfected with shRNA plasmid for SAV1 or siRNA for MST1 and/or MST2. They were stimulated with a full differentiation cocktail containing 10% FBS, 5 nM insulin (Roche, Swiss), 0.5 mM isobutylmethyxanthine (IBMX; Sigma, USA), and 1 µM dexamethasone (Sigma, USA). After 2 d, the culture media was changed to media containing 10% FBS and 5 µg/mL of insulin for 2 d, and then maintained in media containing 10% FBS for 8 d. At 12 d of induction, cells were fixed with 4% formaldehyde for 1 h and then stained with 0.35% Oil Red O dye (Chemicon, USA) overnight at 37°C. The cells were washed twice with PBS and once with 60% isopropanol and then photographed. For quantification, Oil Red O stain was extracted with 100% isopropanol for 20 min and the absorbance at 520 nm was measured.
