# Correction: Mammalian Ste20-Like Kinase and SAV1 Promote 3T3-L1 Adipocyte Differentiation by Activation of PPARγ

**DOI:** 10.1371/journal.pone.0119098

**Published:** 2015-03-18

**Authors:** The PLOS ONE Staff

In the Funding section, the second grant number from the funder National Research Foundation of Korea is listed incorrectly. The correct grant number is: 2009-0084887. Please view the correct funding information here: This work was supported by a research grant (2011-0006216) and a research grant (2009-0084887) to Dr. Lee from National Research Foundation of Korea (NRF) funded by the Korean government (MEST). The funders had no role in study design, data collection and analysis, decision to publish, or preparation of the manuscript.
